# Am I seeing my hand? Visual appearance and knowledge of controllability both contribute to the visual capture of a person's own body

**DOI:** 10.1098/rspb.2012.0750

**Published:** 2012-05-30

**Authors:** Nobuhiro Hagura, Satoshi Hirose, Michikazu Matsumura, Eiichi Naito

**Affiliations:** 1 ATR Brain Information Communication Research Laboratory Group, Kyoto 619-0288, Japan; 2 Institute of Cognitive Neuroscience, University College London, London WC1N 3AR, UK; 3 Graduate School of Human and Environmental Studies, Kyoto University, Kyoto 606-8501, Japan; 4 CiNet, National Institute of Information and Communications Technology, Kyoto 619-0288, Japan; 5 Graduate School of Medicine, Osaka University, Osaka 565-0871, Japan

**Keywords:** body perception, multisensory integration, vision, kinaesthesia, proprioception

## Abstract

When confronted with complex visual scenes in daily life, how do we know which visual information represents our own hand? We investigated the cues used to assign visual information to one's own hand. Wrist tendon vibration elicits an illusory sensation of wrist movement. The intensity of this illusion attenuates when the actual motionless hand is visually presented. Testing what kind of visual stimuli attenuate this illusion will elucidate factors contributing to visual detection of one's own hand. The illusion was reduced when a stationary object was shown, but only when participants knew it was controllable with their hands. In contrast, the visual image of their own hand attenuated the illusion even when participants knew that it was *not* controllable. We suggest that long-term knowledge about the appearance of the body and short-term knowledge about controllability of a visual object are combined to robustly extract our own body from a visual scene.

## 1. Introduction

Seeing one's own body is a unique experience. As a visual stimulus, we see it like any other physical object, but at the same time, we feel that it belongs to us, and we feel that what we see coincides with our somatosensory (kinaesthetic) perception. It is well known that vision is a dominant source of information when perceiving the position of one's own hand [1–3]. However, it still remains uncertain how we effectively extract hand-related visual information from unrelated information in the same visual scene. Even though this process is important for using visual information into the positional estimate of the hand, it has rarely been investigated.

One of the strategies for visual detection of the hand may be to search for a visual object whose location is spatially congruent with the kinaesthetically perceived hand position. Nevertheless, not all of the visual objects located near the hand are perceived as one's own hand [4]. In this study, we investigate the non-spatial cues that are used to assign visual information to one's own hand.

Compared with other natural objects, human body parts have unique visual features (*visual appearance of the body*), which are known to be processed by specialized brain areas [5]. Also, it is proposed that the experience of having control over a visible object plays an important role in feeling the ownership of the body (*knowledge of controllability* [6]). We hypothesized that these two factors jointly contribute to the visual detection process of one's own hand.

To test this hypothesis, we examined the visual attenuation effect of kinaesthetic illusions. Vibration of a wrist muscle tendon elicits the sensation of wrist movement even in the absence of actual movement [7–10]. When participants see their motionless wrist during the illusion, the illusory sensation of movement weakens [11], reflecting the incorporation of visual hand information into the positional estimate of the hand [3]. Thus, by evaluating what kind of visual stimuli attenuate the kinaesthetic illusion, we can clarify the characteristics of visual stimuli that are recognized as information of one's own hand.

**Figure 1.**
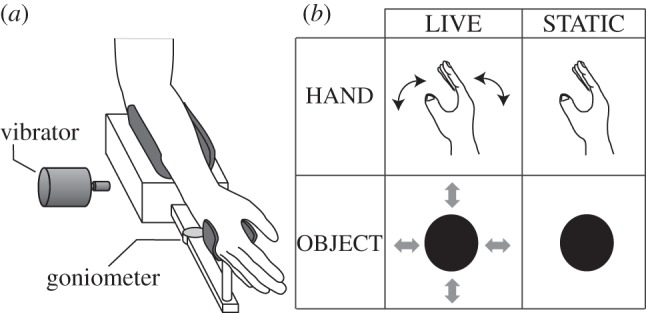
(*a*) Experimental arm fixation. When vibratory stimuli were applied to the tendon, the robotic joint was fixed, so that wrist movement was prevented. During training, a robotic joint allowed participants to move their wrist. The movement was recorded by a goniometer attached to the robotic joint. (*b*) Visual stimuli presented during tendon vibration. Participants viewed one of the four visual stimuli, which all remained stationary throughout the task. The arrows indicate the possible effects participants can induce to the visual stimuli by moving their hand, when the hand is unfixed. Note that the participants were informed of the conditions LIVE or STATIC before each trial (see §2).

In the present study, participants viewed either the motionless image of their hand or that of an object (*visual appearance* factor) while experiencing the kinaesthetic illusion (figure 1*b*). Each of the visual stimuli was presented in two different conditions. In one condition, participants knew that the image (hand/object) were controllable through hand movement. In the other condition, they were informed that the image was uncontrollable (*knowledge of controllability* factor). The attenuation rate of the kinaesthetic sensation under each visual condition was examined.

## 2. Methods

### (a) Participants

A total of 13 right-handed healthy male (nine) and female (four) volunteers (age: 21–32) participated in the study. Procedures were approved by the Kyoto University ethics committee.

### (b) Experimental procedure

The right wrist *extensor carpi ulnaris* (ECU) muscle of the participants was stimulated by vibrations, inducing illusory flexion movement of the right hand. During stimulation, participants were subject to different visual conditions. They sat comfortably in a chair with the semi-pronated right arm placed in an experimental device (figure 1*a*). The wrist was positioned at a 0° angle and immobilized with a brace during stimulation. Vibration was applied with a mechanical stimulator and the frequency was set to 80 Hz (amplitude: 3.5 mm; ILLUSOR, Umihira Ltd., Kyoto, Japan [3]).

The visual images were presented on a head-mounted display (Z800 3DVISOR, eMagin, New York, NY). While wearing the display, participants faced towards the actual location of the hand. Two kinds of images were displayed: a hand (HAND) and an object (OBJECT). For each visual image, there were two conditions: live (LIVE) and static (STATIC). Therefore, the experiment was conducted in a 2 (hand or object) × 2 (live or static) factorial design (figure 1*b*).

In the HAND–LIVE condition, participants saw their own vibrated right hand on the display through an online video-camera (DSP 268X, Mother Tool, Nagano, Japan). The video-camera was set up 45 cm above their hand, which allowed the position and the size of the hand image to coincide approximately with the position and size of their hand when actually seen. The video image captured the whole hand (from the wrist joint to the tips of the fingers). In the HAND–STATIC condition, a static image of the participant's hand recorded prior to the experiment in the same position as in the HAND–LIVE condition was presented. The set-up of the experiment and the example image of the hand conditions are presented in the electronic supplementary material section.

In the OBJECT–LIVE condition, participants were presented with a black disc that changed its radius linearly with the flexion angle of the right hand. The wrist angle was continuously measured by the goniometer attached to the experimental device. The disc expanded when the wrist was flexed and shrank when it was extended. Finally, in the OBJECT–STATIC condition, participants viewed a picture of a static black disc whose size corresponded to the disc size at 0° wrist angle in the OBJECT–LIVE condition.

Note that the participant's wrist was fixed at 0° in all visual conditions during the tendon stimulation, so that the visual image was in fact motionless in all conditions regardless of the stimulus property. In order to inform participants of the stimulus property in each visual condition, participants performed a simple motor task just before the vibration task. In this task, participants moved their wrists while viewing the same type of visual stimulus as was presented after this motor task. The wrist was unfixed and they were able to move the wrist in the flexion–extension axis. A tone was repeatedly presented in 1 Hz, and the participants made flexion movement for the first tone and extension movement for the next tone, and kept performing this cyclic flexion–extension movement for 30 seconds. Therefore, only during this period, participants were able to extract the information about the stimulus property by recognizing whether the visual image moves synchronously with their wrist movement (LIVE) or not (STATIC).

In addition to the earlier-mentioned four visual conditions, we introduced the CLOSE condition, in which participants received the vibratory stimulation with their eyes closed. They performed the pre-vibration motor task also for this condition. This condition served as a baseline for the strength of illusion experienced under the different visual conditions.

Each of the five conditions was presented as a block of four trials. The order of the conditions was randomized across participants. In each trial, the tendon was stimulated for 20 s. After that, participants were asked to quantify the degree of perceived hand flexion with a score from 0 to 10 (illusion score). A score of 10 indicated that they had experienced an illusory movement of maximally flexed wrist angle, while a score of 0 indicated that they felt no illusory movement at all [3].

### (c) Training to control the OBJECT–LIVE stimulus

**Figure 2.**
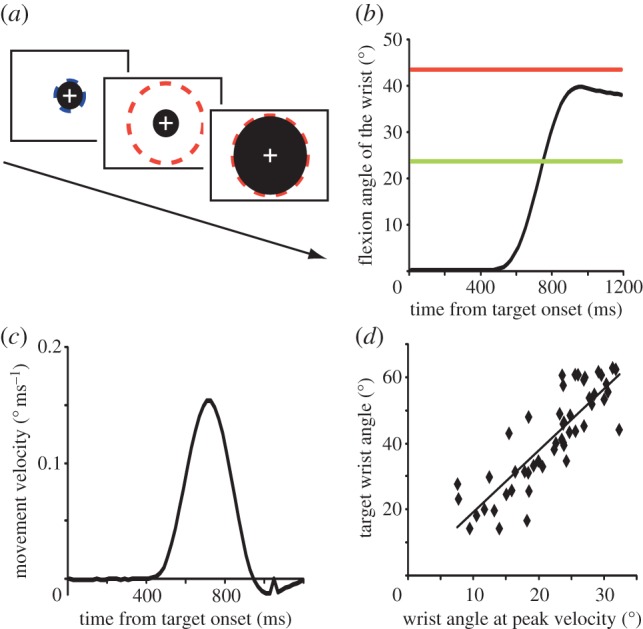
(*a*) Trial task sequence during training to control the OBJECT–LIVE stimulus. Participants controlled the size of the black disc to change from the start size (blue dotted line) to the target size (red dotted line) through ballistic wrist flexion movements. (*b–d*) Movement profile of a trial from a representative participant in the third session of the training. (*b*) The trajectory of angle displacement in a trial is plotted against time from the target onset (black). Red horizontal line indicates the target angle of this trial, and green indicates the wrist angle at the point of peak velocity (angle at peak velocity of (*c*)). (*c*) Data from the same trial is displayed as change in the angular velocity across time. The bell-shaped profile of the movement velocity indicated the successful generation of a ballistic wrist movement. (*d*) The relationship between the target wrist angle (red line in (*b*)) and the peak velocity wrist angle (green line in (*b*)). Each black dot represents a single trial. These variables were well correlated, indicating accurate and stable motor planning across trials.

Prior to the assessment of the illusion, participants underwent a training to control the size of a black disc on the screen with their right wrist (disc in the OBJECT–LIVE condition, figure 2*a*). This was done to familiarize participants with the stimulus controllability. More specifically, participants learned the mapping between the wrist flexion angle and the size of the disc in the OBJECT–LIVE condition through this training, namely, how the size of the visual stimulus changes in relation to their wrist angle.

Each trial started with the appearance of a blue hollow circle whose size corresponded to the 0° extended position of the wrist. After the participants adjusted the size of the black disc to the size of the blue circle (start disc), a red hollow circle appeared, indicating the target disc, and participants flexed their wrist in a ballistic manner to match the black disc with the target disc. The target disc was presented for one second. Then the start disc reappeared for the next trial. Three training sessions, each consisting of 50 trials, were conducted. The size of the target disc was randomized across trials, in a range corresponding to 15–65° wrist flexion angle. Wrist angular data recorded with the goniometer were fed to a personal computer for online control of the LIVE–OBJECT stimulus and saved for offline analyses (50 Hz; USB-6210, National Instruments, Austin, TX). One participant was excluded from the offline movement data analysis owing to a recording failure of the wrist movement data.

## 3. Results

### (a) Movement data of wrist control during the training

Participants successfully performed ballistic wrist movements during the training, as was evident by the bell-shaped movement velocity profile [12] (figure 2*b,c*). Once the participants have fully learned to control the stimulus, the control planning (feed-forward component) should be accurate. To confirm this was the case for controlling the object here, in each trial we first determined the wrist angle at maximum movement velocity (peak velocity angle). The movement profile before the peak velocity reflects the feed-forward component of the movements (component of planned movement), rather than the component of final adjustment. Thus, if the peak velocity angle changes linearly according to the target angle, accurate and stable movement planning can be expected. On the basis of this idea, we defined the correlation coefficient between the peak velocity angle and the target angle as ‘index of controllability’ (figure 2*d*). This was computed from each of the three training session.

The correlation coefficient between peak velocity angle and the target angle (index of controllability; figure 2*d*) was consistently high across all sessions and participants (average *R*: 0.85, s.d. = 0.05). No learning effect was observed across sessions (*F*_2,11_ = 0.39, *p* = 0.68, partial *η*^2^ = 0.04). This shows that participants rapidly learned to control the visual object, and grasped the controllability of the object during the course of training.

### (b) Illusion attenuation under different conditions

**Figure 3.**
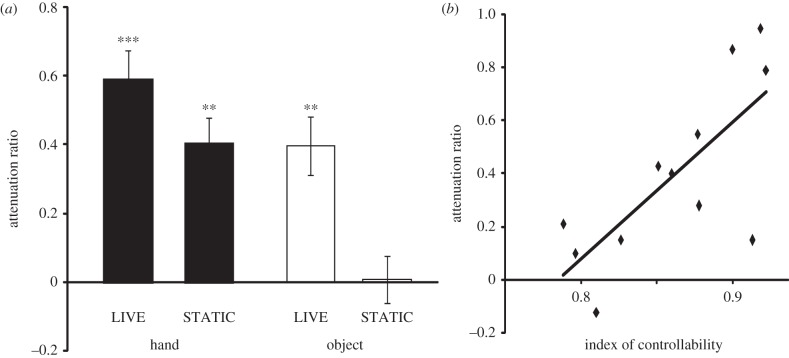
(*a*) Rate of illusion decrease from the close condition under each visual condition (attenuation ratio) averaged across participants. Error bars indicate the s.e. of the mean value across participants. ***p < 0.001, **p < 0.005. (*b*) Relationship between attenuation ratio and controllability index in the OBJECT–LIVE condition.

The attenuation effect on the illusion was evaluated for each visual condition by calculating the ratio of the average illusion score reduction compared with the illusion score in the control (CLOSE) condition (attenuation-ratio = (close − visual)/close)*.* Significant attenuation of the illusion was observed in the HAND–LIVE condition (one-sample *t*-test, d.f. = 12, *t* = 6.6, *p* < 0.001 corrected with Bonferroni, *d* = 1.91), the HAND–STATIC condition (*t* = 5.0, *p* < 0.005 corrected, *d* = 1.45) and the OBJECT–LIVE condition (*t* = 4.6, *p* < 0.005 corrected, *d* = 1.29), but not in the OBJECT–STATIC condition (*t* = 0.1, *p* = 0.91 uncorrected, *d* = 0.03) (figure 3*a*). A two-factorial (*visual appearance*, hand or object × *knowledge of controllability*, live or static) ANOVA on the attenuation ratio revealed main effects for both factors: the *knowledge of controllability* (*F*_1,12_ = 12.7, *p* < 0.005, partial *η*^2^ = 0.52) and the *visual appearance* (*F*_1,12_ = 18.6, *p* < 0.001, partial *η*^2^ = 0.61). No interaction between the factors was observed (*F*_1,12_ = 2.3, *p* = 0.16, partial *η*^2^ = 0.16). To ensure that the result is neither an artefact of transforming the illusion scores into ratio of reduction from the CLOSE, nor an artefact of nonlinearity between wrist angle of perceived movement with the illusion score, we also performed two additional analyses: (i) the same analysis for the raw illusion scores and (ii) a non-parametric ANOVA (Friedman test) for the raw illusion scores. These additional analyses showed an identical pattern of results, namely, significant main effects for both factors without any significant interaction. Therefore, the current findings were not affected by the normalization procedure we have performed (see electronic supplementary material).

In the OBJECT conditions, significant attenuation of the illusion was observed only when participants knew that the object was controllable (LIVE). We further tested whether the ability to precisely control the object is reflected in the attenuation ratio. Specifically, we tested whether there is a relationship between the attenuation ratios in the OBJECT–LIVE condition with the index of controllability during the training of controlling the disc (see §3*a*). Index of controllability in the third (last) session was used, because this would most closely represent the motor performance level at the point of assessment of the illusion. A significant correlation was found between the two variables (d.f. = 10, *R* = 0.73, *t* = 3.4, *p* < 0.01; figure 3*b*). This indicates that the solidity of the visuomotor association learned *before* the experiment is reflected as the amount of incorporation of the static visual stimulus into the positional estimate of the hand. The same analysis was conducted for the OBJECT–STATIC condition, in which the features of the visual stimuli are identical to that in the OBJECT–LIVE condition. The results confirmed that this relationship does not hold when participants knew that the presented object was uncontrollable (d.f. = 10, *R* = 0.12, *t* = 0.4, *p* = 0.71). Moreover, the correlation did not reach significance for any of the hand conditions (HAND–LIVE: *R* = 0.24, *t* = 0.78, *p* = 0.45; HAND–STATIC: *R* = 0.38, *t* = 1.31, *p* = 0.21). The specificity of this relationship between the motor performance and the attenuation of the illusion observed during the OBJECT–LIVE condition demonstrates that this finding is not simply reflecting the precision of general visuomotor transformation ability.

## 4. Discussion

By assessing the effects of visual information on the illusory displacement of the hand, we demonstrated that the knowledge of having control over the visual stimulus and the visual features of a human hand both jointly contribute to perceiving the visual input as related to one's own hand.

In the present study, visual attenuation of kinaesthetic illusion was used as a signature for visual information to be processed as from one's own hand. We have shown in a previous study that viewing the non-moving vibrated hand attenuates the kinaesthetic illusion, whereas viewing the contralateral non-vibrated hand does not [3]. This indicates that only visual information of a hand relevant to the kinaesthetic input attenuates the illusion. Moreover, in the object conditions of the present study, we found significant attenuation of the illusion only in the LIVE condition, even though the visual features of the stimulus were identical with the STATIC condition (figure 3*a*). These results show that only visual stimuli containing online positional information relevant to the kinaesthetic input, not the low level visual features, modulate the illusion. Therefore, the attenuation of kinaesthetic sensation reflects the combinability of the visual stimulus with the kinaesthetic input, and thus can be used as the index of assignment of visual positional information to one's own hand.

Even though the visual stimulus was set to minimize the differences between the live and the static conditions, there still may be a possibility that there was an observable difference between the two stimuli, such as by subtle unconscious finger movements or by fluctuation of streaming image noise. But in any case, before the application of the vibration, participants *knew* what the stimulus property was (live or static) by the movement they have made before each trial (see §2). So there was no need for them to further detect the property of the stimulus. Therefore, the subtle differences in the visual image may have strengthened the participants' perceived context of the controllability (live or static), but we believe that the effect induced by the context should not qualitatively differ from what we have reported here.

For the OBJECT conditions, significant attenuation of illusion was selectively observed in the live condition, and the attenuation ratio significantly correlated with the individual performance in the feed-forward control of the stimulus (black disc). Note that the motor performance was measured *before* assessment of the illusion and the hand was fixed during the vibration; the participants could not monitor the strength of visuo-motor association online during the illusion. This correlation thus indicates that the association between vision and kinaesthetic information of hand for the object control (internal-model [13,14]) acquired during training is used for the estimation of hand position. Variance in accuracy of this model may have lead to vary the usage of the stationary visual information for evaluating the state of the hand (attenuate the kinaesthetic sensation) [1,2]. Importantly, when the object was uncontrollable, illusion was not attenuated, and the correlation between motor performance and the attenuation rate of illusion was not observed. This implies that the acquired internal model is loaded only when the participants knew that the viewed object is controllable. It has been shown that different internal models for hand control are flexibly recruited when manipulating objects that require different control dynamics to normal [15–17]. Our result further shows that an appropriate internal model is selected depending on the context, and used not only for motor control but also for visual processing of estimating the static position of one's own hand. The specific incorporation of visual information as related to one's own hand for a controllable object (OBJECT–LIVE) fits with the notion that a hand-held object can be rapidly assimilated into the body representation, but not when it is held by a device that the participants are unfamiliar of [18]. Neurons in the parietal cortex may contribute to this ability; a study on macaques has shown that neurons in the parietal cortex, whose visual receptive field is usually restricted to a space near the hand, expand their visual receptive field to include the tool-use space, but only when the monkey is ready to use the tool [19].

When the stimulus was an image of the hand, the controllable stimulus (HAND–LIVE) attenuated the illusion, as has been shown previously [3,11]. However, in contrast to the object conditions, the illusion was also significantly attenuated even when participants knew that the hand image was uncontrollable (HAND–STATIC). This automatic coupling between visual hand features and the kinaesthetic hand information corroborates studies that show that even a schematic drawing of a hand unrelated to one's own hand can enhance tactile spatial discrimination [20,21]. Considering these evidences, we suggest that a visual object with hand features is directly assigned to one's own hand without assessing its controllability. Because in daily life the vision of one's own hand is always associated with the somatosensory inputs of the hand, this linkage in our brain might have been established through life-long associative learning. Visual body part information is processed in humans distinctively in the extrastriate visual areas (extastriate body area and fusiform body area) [5]. It has been shown that a locus nearby extrastriate body area, part of lateral occipital cortex, preferentially activates for visual stimuli of hand [22]. Interestingly, in this area, it is found that the neuronal activity pattern is similar between when visual information of hands is presented, and when visual information of daily hand-used objects is presented [23]. Processing visual tool information in the same neuronal representation as the hand is implemented through an extended period of experience. This may support our claim that the daily visual–kinaesthetic association is important to induce the brain to extract the visual feature of the hand (tool) as something related to one's own hand. If so, the visual areas coding visual features of hand should also receive motor/kinaesthetic information from the hand. In fact, recent neuroimaging studies have found activations in the occipital and temporal cortices during motor control, even without any visual input [24,25]. More precisely, the posterior part of superior temporal sulcus, which can also be activated by visual information of body parts, is reported to change its activation level depending on the congruency of the visual hand motion to one's own hand action [26]. These neuroimaging results suggest that the locus processing visual features of a body part might also be involved in the sensorimotor processing of the same. Taken together, neuronal architectures in the visual cortices that are involved in the processing of both visual feature information of the hand and kinaesthetic hand information may contribute to linking the two, allowing visual information of hand to directly affect the positional estimate of the hand [27].

The present study revealed that the factors’ *visual appearance* and *knowledge of controllability* both contribute to process of extracting visual hand information. The illusion attenuated the most when participants viewed the live image of their own hand (HAND–LIVE), as is the case in natural situations, indicating that the two factors work together for the visual detection of one's own hand. Controllability of the visual stimulus in the OBJECT–LIVE condition was acquired in a short training period. In contrast, the association between the visual features of one's own hand and its kinaesthetic information as proposed earlier has probably been established through life-long learning. It has been postulated that humans combine multiple motor learning systems, which adapt to the environment at different timescales (fast and slow learning systems), in order to cope with the environmental changes also occurring at different time scales [28,29]. By analogy with this combination strategy, our data may imply that two different cues acquired in different timescales are combined to efficiently and robustly detect own hand information in a noisy visual environment.

In contrast to previous studies, which focus on sensory integration mechanisms of visual and other sensory information from the body [1,2,30], our study highlights the process *prior* to that integration process—the process of extracting relevant visual information for computing one's own body position. We propose that our percept of a ‘unified body image’ is maintained not only by the combination of bottom-up multisensory inputs, but also by top-down knowledge about whether the given sensory inputs are combinable. Before processing visual information as from our own body, we check what it looks like, and verify whether it can be controlled.
